# Endophytic Fungi Activated Similar Defense Strategies of *Achnatherum sibiricum* Host to Different Trophic Types of Pathogens

**DOI:** 10.3389/fmicb.2020.01607

**Published:** 2020-07-24

**Authors:** Xinjian Shi, Tianzi Qin, Hui Liu, Man Wu, Juanjuan Li, Yansong Shi, Yubao Gao, Anzhi Ren

**Affiliations:** College of Life Sciences, Nankai University, Tianjin, China

**Keywords:** *Achnatherum sibiricum*, endophyte, jasmonic acid, pathogens, trophic type

## Abstract

It is well documented that *Epichloë* endophytes can enhance the resistance of grasses to herbivory. However, reports on resistance to pathogenic fungi are limited, and their conclusions are variable. In this study, we chose pathogenic fungi with different trophic types, namely, the biotrophic pathogen *Erysiphales* species and the necrotrophic pathogen *Curvularia lunata*, to test the effects of *Epichloë* on the pathogen resistance of *Achnatherum sibiricum*. The results showed that, compared to *Erysiphales* species, *C. lunata* caused a higher degree of damage and lower photochemical efficiency (*Fv/Fm*) in endophyte−free (E−) leaves. Endophytes significantly alleviated the damage caused by these two pathogens. The leaf damaged area and *Fv/Fm* of endophyte−infected (E+) leaves were similar between the two pathogen treatments, indicating that the beneficial effects of endophytes were more significant when hosts were exposed to *C. lunata* than when they were exposed to *Erysiphales* species. We found that *A. sibiricum* initiated jasmonic acid (JA)−related pathways to resist *C. lunata* but salicylic acid (SA)–related pathways to resist *Erysiphales* species. Endophytic fungi had no effect on the content of SA but increased the content of JA and total phenolic compounds, which suggest that endophyte infection might enhance the resistance of *A. sibiricum* to these two different trophic types of pathogens through similar pathways.

## Introduction

Plant diseases are consistently among the important factors restricting the quality and yield of crops. It is estimated that plant diseases, 70∼80% of which are caused by pathogenic fungi, lead to an average loss of 10∼15% of the world’s major cash crops and direct economic losses of hundreds of billions of dollars each year (Strange and Scott, 2005; Kang, 2010). Depending on the ways in which nutrients are obtained from host cells, plant pathogenic fungi are classified as biotrophs, necrotrophs, or hemibiotrophs (Niks and Marcel, 2009). Biotrophic fungi obtain nutrients only from living host cells; necrotrophic fungi kill the host cells and then extract nutrients from the dead cells for their own growth and reproduction, and hemibiotrophic fungi grow like biotrophs in the initial stage of host cell infection and then turn into a necrotrophic phase (Perfect and Green, 2001; Niks and Marcel, 2009; Kou and Naqvi, 2016).

Phytohormones, such as jasmonic acid (JA) and salicylic acid (SA), are the central defense signaling molecules that regulate the defense responses of plants to pathogens. In *Arabidopsis thaliana*, Thomma et al. (1998) found that the *npr1* mutation, which blocked SA signaling, resulted in greater susceptibility to the biotrophic fungus *Peronospora parasitica* but had no effect on resistance to the necrotrophic fungus *Alternaria brassicicola*. Conversely, the *coi1* mutation, which blocked JA signaling, severely compromised resistance to the necrotrophic fungus *A. brassicicola* but had no effect on resistance to *P. parasitica*. Such results indicated that plants may initiate different defense mechanisms in response to different trophic types of pathogenic fungi, with SA−dependent defenses acting against biotrophs and JA−dependent responses acting against necrotrophs (Glazebrook, 2005). Both SA and JA can enhance the activity of enzymes in the phenylpropane pathway in plants and induce the synthesis of phenolic compounds (Neerja et al., 2013; Islam et al., 2019b). Plant phenolics are involved in disease resistance mechanisms in a variety of pathosystems, and phytohormones act as significant regulatory factors of disease tolerance (Cheynier et al., 2013; Islam et al., 2019a).

Endophytic fungi of genus *Epichloë* form symbiotic relationships with cold−season grasses (Tanaka et al., 2012). Endophytes obtain nutrients from host plants (Clay and Schardl, 2002) and in return might promote host growth (Malinowski et al., 1998; Li et al., 2012) and enhance the tolerance of host plants to abiotic and biotic stresses such as drought (Bouton et al., 1993; Tanaka et al., 2012) and herbivory (Tanaka et al., 2005; Baldauf et al., 2011; Qin et al., 2016). In addition, *Epichloë* can affect the disease resistance of host plants, but the direction of influence is not consistent. To date, positive, neutral, and even negative effects have been reported. For example, *Epichloë* enhanced the resistance of *Festuca arundinacea* and *Lolium perenne* hosts to *Rhizoctonia zeae* (Christensen, 1996; Pańka et al., 2013b) but had no significant effects on the resistance of *Festuca pratensis* to *Puccinia graminis* or *Fusarium oxysporum* (Welty et al., 1993; Trevathan, 1996; Pańka et al., 2011). Wäli et al. (2006) even found that *Lolium pratense* infected by *Epichloë* was more sensitive than uninfected *L. pratense* to *Typhula ishikariensis*. We hypothesize that the inconsistencies in the effects of *Epichloë* on the resistance of host grasses are related not only to the species of symbiont but also to the trophic mode of pathogenic fungi.

It is well known that endophyte infection can improve herbivore resistance of the host grasses due to production of alkaloids (Clay and Cheplick, 1989; Ball et al., 1997; Bush et al., 1997; Schardl et al., 2013). However, alkaloids are not likely directly associated with fungal pathogen resistance (Siegel and Latch, 1991; Holzmann et al., 2000; Schardl et al., 2013; Bastías et al., 2017). Then, how does endophyte infection improve pathogen resistance of the host? The pioneering research by Malinowski et al. (1998) found that *Epichloë* endophytes increased the production of phenolic compounds in roots of *F. arundinacea*, and similar results were reported in *L. perenne* (Pańka et al., 2013a; Pierre et al., 2015). Thus, the elevated levels of total phenolic compounds might be correlated with plant resistance to pathogenic fungi. Recently, Bastías et al. (2018a, b) found that symbiotic plants had lower concentrations of SA than their non-symbiotic plants, and SA/JA treatment decreased the endophyte-conferred resistance against aphids. These results indicated that SA/JA might play a critical role in regulating the endophyte-conferred resistance against herbivores. Therefore, studies on this subject about SA/JA involved in pathogen resistance of *Epichloë*-infected grasses are very limited (Wang et al., 2016; Guo et al., 2019).

*Achnatherum sibiricum* is a perennial, sparse bunchgrass that is widely distributed in Northeast China and is usually colonized by *Epichloë* endophytes with high infection rates (86–100%) in natural habitats (Wei et al., 2007; Zhang et al., 2009). Within the genus *Achnatherum*, two other species, *Achnatherum inebrians* and *Achnatherum robustum*, have been reported to be infected by *Epichloë* endophytes. Both are notorious for their narcotic properties in livestock and hence are named as “drunken horse grass” and “sleepy grass,” respectively (Petroski et al., 1992; Bruehl et al., 1994). *Achnatherum inebrians* can be infected by *Epichloë gansuensis* and *Epichloë inebrians* (Chen L. et al., 2015), and *A. robustum* by *Epichloë funkii* (Moon et al., 2007). As for *A. sibiricum*, it can harbor two different *Epichloë* species, *Epichloë sibirica* and *E. gansuensis*. The phenomenon of double infections by both *Epichloë* species in the same plant has not been observed in *A. sibiricum* (Zhang et al., 2009; Li et al., 2015). Both *E. sibirica* and *E. gansuensis* improved the growth and competitive ability of *A. sibiricum* (Li et al., 2016; Zhou et al., 2019), and their main metabolites were also similar (our unpublished data). According to many years of observations in our laboratory, *A. sibiricum* exhibits no obvious herbivore deterrence, but its pathogen damage is usually less serious than in most neighboring plants of other species. In this study, *Epichloë*−infected and *Epichloë*−free *A. sibiricum* were used as plant materials, and a biotrophic fungus, *Erysiphales* species (powdery mildew), and the necrotrophic fungus, *Curvularia lunata* were selected as pathogens. The following questions were addressed: (1) Can *Epichloë* improve the resistance of *A. sibiricum* to pathogens? (2) Is the influence of endophytic fungi on the disease resistance of host plants related to the trophic types of pathogens? (3) What is the possible mechanism?

## Materials and Methods

### Plant and Pathogenic Fungi Materials

Seeds of *A. sibiricum* were collected from the *Stipa baicalensis* sampling area of Yimin in the National Hulunbuir Grassland Ecosystem Observation and Research Station of China (119.669°E, 48.493°N). Detection of endophytic fungus was carried out on the seeds by the aniline blue–lactic acid staining method (Latch et al., 1984), and their endophyte infection rates were 100%. Endophyte−free (E−) seeds were obtained from endophyte−infected (E+) seeds by high−temperature treatment (60°C) for 30 days (Li et al., 2010). Earlier work in our laboratory showed that the high−temperature processing had no significant effects on the seed germination rate (Li et al., 2010) and was an effective disinfection method for *A. sibiricum* (Ren et al., 2011; Li et al., 2012). In the previous study of our laboratory, the effect of different species of endophytes on fungal disease resistance of *A. sibiricum* was studied, and the results showed that the resistance of *A. sibiricum* was not affected by endophyte species (Niu et al., 2016). Therefore, in this study, we did not discriminate endophyte species, and the seeds were infected by either *E. sibirica* or *E. gansuensis*.

The seeds were surface sterilized with 2% NaClO solution for 5 min, flushed with sterile water for 3 times, and then placed on potato dextrose agar (PDA; Sangon Biotech Company, Shanghai, China) in the dark at 25°C. After 4 weeks, only *E. sibirica* or *E. gansuensis* was isolated from E+ seeds, but no microbe was isolated from E− seeds. Thirty sterilized seeds were evenly spread in each pot (200 mm in diameter and 220 mm in depth) filled with sterilized vermiculite. After 45 days, the endophyte status of all plants was checked microscopically by examining the upper epidermis of leaf sheaths stained with lactophenol containing aniline blue (Latch and Christensen, 1985). The endophyte−infected proportions of E+ plants were 100%, and endophyte−free proportions of E− plants were zero. Twenty plants of approximately equal size (approximately 15 cm high) were maintained in one pot.

*Erysiphales* species was collected from the diseased leaves of *A. sibiricum*. To purify the pathogen, we cut from the margins of actively growing fungal colonies and immediately placed in petri dishes containing 1% (wt/vol) distilled water agar and 8.5 mM benzimidazole (Wang et al., 2014). A single colony of *Erysiphales* species was transferred to inoculate a healthy plant, and this process was repeated three times. *Curvularia lunata* was purchased from the Agricultural Culture Collection of China. A spore suspension of *C. lunata*, which was cultured on PDA for 15 days at 28°C, was prepared by washing the hyphae of the pathogenic fungus with sterile water (containing 0.02% Tween 20) and filtering with double layers of sterile gauze. The concentration of the pathogen spore suspension counted by hemocytometer was 1.4 × 10^6^ spores/mL, and the spore germination rate was 87%.

### Experimental Design

The experiment followed a randomized block design with two factors. The first factor was the *Epichloë* endophyte status, including E+ and E−. The second factor was the inoculation of pathogenic fungi, including the following three treatments: control (CK), *C. lunata*, and *Erysiphales* species. Each combination was replicated 10 times, yielding a total of 60 pots. The experiment was conducted in the greenhouse at Nankai University, Tianjin, China. Plants were subjected to ambient light, and the room temperature was 20–30°C. During the experiment, each pot was watered once a week with one-half strength Hoagland nutrient solution. The experiment began on November 1, 2018, and lasted for 3 months.

### Inoculation of Pathogenic Fungi

The leaves were inoculated with *C. lunata* by spraying them with the spore suspension until liquid dripped from them, and CK was sprayed with sterile water containing 0.02% Tween 20. For the inoculation of *Erysiphales* species, the conidia collected from the cultured plants were blown uniformly onto E+ and E− plants with a hair dryer according to Li’s method (Li et al., 2018). After inoculation, all tested plants were transferred to transparent storage boxes, where high humidity was maintained to promote the disease on leaves.

### Observation of Leaf Damage

Fully expanded diseased leaves collected randomly 0, 3, and 7 days after pathogen inoculation were examined by scanning electron microscopy (Quanta 200 scanning electron microscope, FEI; Portland, Oregon, United States) (Becker et al., 2016), and the tissue structure and mycelia on the blade surface were observed.

On the seventh day, 10 plant leaves were randomly sampled and stained by trypan blue under each treatment (Michael et al., 2018). The damaged leaf areas were stained blue, whereas the healthy areas were colorless. These stained leaves were photographed one by one with a digital camera. Then, we calculated the proportion of the trypan blue–stained area of leaf photos using ImageJ software (Taheri and Kakooee, 2017).

### Measurement of Response Variables

After 7 days of pathogen infection, *Fv/Fm*, the maximum quantum efficiency of photosystem II in the dark−adapted state, was recorded on the disease spot with a Fluorpen FP 110 handheld fluorometer (Pneumatic System International; Brno, Czech Republic), and *Fv/Fm* of CK leaves on the same site was also recorded.

Freeze−dried leaf samples of 0.3–0.5 g (fresh weight) were taken for quantification of SA and JA. The SA content was quantified using high−performance liquid chromatography (Waters 1500−series; Micromass UK Ltd., Manchester, United Kingdom) on a C18 reverse−phase column following Wang’s method with modification (Wang et al., 2016). The JA content was quantified by enzyme−linked immunosorbent assay (ELISA) (JA ELISA Kit; Shanghai Yingxin Laboratory, Shanghai, China).

Approximately 0.3 g leaf samples (dry weight) were taken for qualification of total phenolic compounds. The total phenolic compounds content was determined by Folin−Ciocalteu colorimetry with Shimadzu UV-1800 double-beam spectrophotometer (Shimadzu; Kyoto, Japan) (Chen L. Y. et al., 2015).

### Data Analyses

All data analyses were performed with SPSS software (version 22.0; IBM, Armonk, New York, United States). Two−way analysis of variance was used to analyze the effects of endophyte infection and pathogen inoculation on all response variables of *A. sibiricum*. The differences among means were compared using Duncan multiple−range test, with significance at *P* < 0.05.

## Results

### Microscopic Observations

**FIGURE 1:**
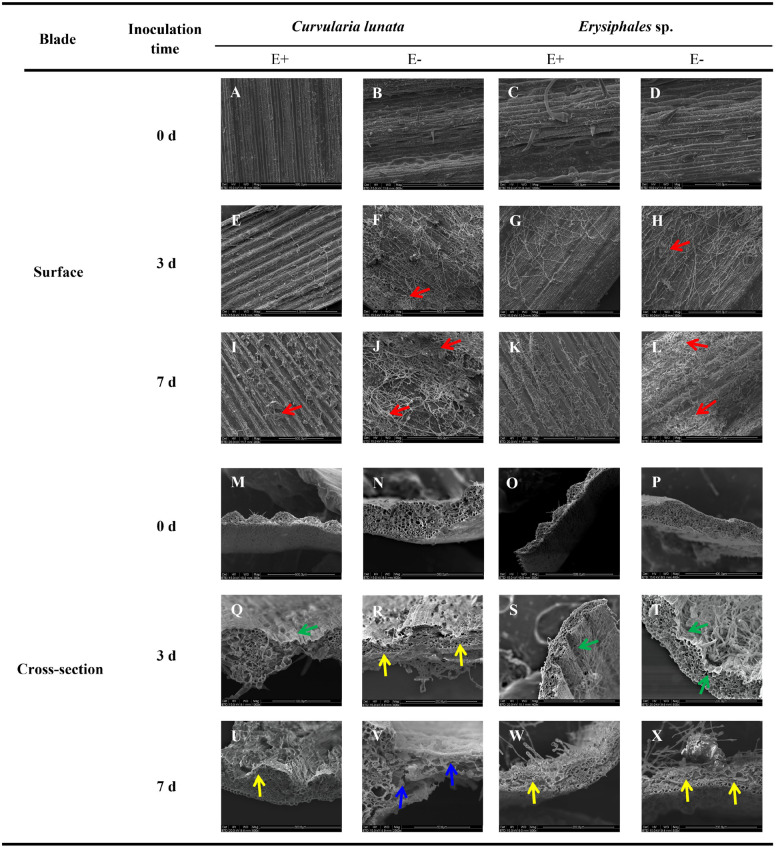
Scanning electron microscopy observation of structures of E+ and E− *A. sibiricum* leaves infected by pathogens. Images A-L indicate the surface of E+ and E- leaves inoculated by *C. lunata* or *Erysiphales* sp. on Day 0 **(A–D)**, 3 **(E–H)**, and 7 **(I–L)**, respectively. Images M-X indicate the cross-section of E+ and E- leaves inoculated by *C. lunata* or *Erysiphales* sp. on Day 0 **(M–P)**, 3 **(Q–T)** and 7 **(U–X)**, respectively. Red arrows indicate infection cushions; green arrows indicate crumpling cell walls; yellow arrows indicate collapsed cell walls, and blue arrows indicate fragmented cell walls.

Before pathogen inoculation, the surface structure was similar in E+ and E- leaves (Figures 1A–D). After pathogen inoculation, scanning electron microscopy revealed that the mycelial density of pathogens in E− leaves was higher than that in E+ leaves at the same time after inoculation of *C. lunata* or *Erysiphales* species (Figures 1E–L). When inoculated with *C. lunata*, infection cushions first appeared on the surface of E− leaves on the third day (Figures 1F,H). For E+ leaves, no infection cushions were observed in response to infection by *C. lunata* until 7 days after inoculation (Figures 1E,G,I,K), and the levels were lower than those observed on E− leaves (Figures 1I,J). When inoculated with *Erysiphales* species, infection cushions appeared on the surface of E− leaves on the third and seventh day (Figures 1H,L), but no infection cushions were observed on E+ leaves (Figures 1G,K).

The degree of damage to leaf cell walls (integrity, crumpling, collapse, and fragmentation) was an important indicator of plant disease resistance. The cross-section structure of E+ and E- leaves was integrity before pathogen inoculation (Figures 1M–P). The damage to leaf structure caused by *C. lunata* inoculation was more severe than that caused by *Erysiphales* species, and the endophytic fungi alleviated the damage caused by both pathogens (Figures 1Q–X). When inoculated with *C. lunata*, crumpling cell walls were observed on the third day (Figure 1Q), and the mesophyll cells began to collapse on the seventh day in E+ leaves (Figure 1U). For E− leaves, the mesophyll cells began to collapse on the third day (Figure 1R), and the whole cross−section cell structure was completely fragmented on the seventh day (Figure 1V). Under inoculation with *Erysiphales* species, crumpling cell walls were observed at the infection site on the third day (Figures 1S,T), and the epidermal cells showed collapse on the seventh day in both E+ and E− leaves (Figures 1W,X), but the degree of crumpling and collapse was more severe in E− leaves.

### Leaf Damage

**FIGURE 2:**
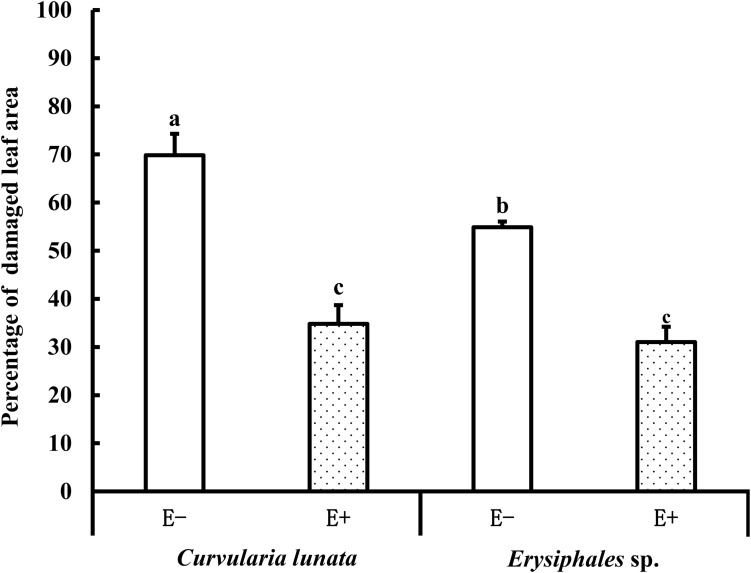
The leaf damage area of E+ and E− *A. sibiricum* leaves infected by pathogens. Different lowercase letters indicate significant differences between treatments (*P* < 0.05). Bars represent mean values ± standard error (SE) (*n* = 10).

The proportions of damaged leaf area were affected by endophytes and pathogens (Table 1 and Figure 2). In E− leaves, *C. lunata* caused a significantly higher proportion of damaged leaf area (70%) than *Erysiphales* species (55%). Endophytes significantly alleviated the damage caused by these two pathogens. In E+ leaves, the proportion of damaged leaf area was similar for the two pathogens. These results indicated that the beneficial effects of endophytes were more significant when hosts were exposed to *C. lunata* than when they were exposed to *Erysiphales* species.

**TABLE 1:** Analysis of variance of the effects of the endophyte (E) and pathogens (P) on the leaf damage area, chlorophyll fluorescence parameters, total phenolic compounds content, and SA/JA content of *A. sibiricum.*

	Leaf damage				Total phenolic					
Treatment	area		*Fv/Fm*		compounds		SA		JA	
	*F*	*P*	*F*	*P*	*F*	*P*	*F*	*P*	*F*	*P*
Endophyte (E)	74.75	**<0.001**	117.088	**<0.001**	43.264	**<0.001**	0.007	0.934	79.429	**<0.001**
Pathogens (P)	7.573	**0.009**	213.647	**<0.001**	108.609	**<0.001**	442.789	**<0.001**	17.663	**<0.001**
E*P	2.692	0.110	52.973	**<0.001**	13.973	**<0.001**	1.151	0.333	1.035	0.370

*Significant P-values (P < 0.05) are shown in bold font.*

### Chlorophyll Fluorometry

**FIGURE 3:**
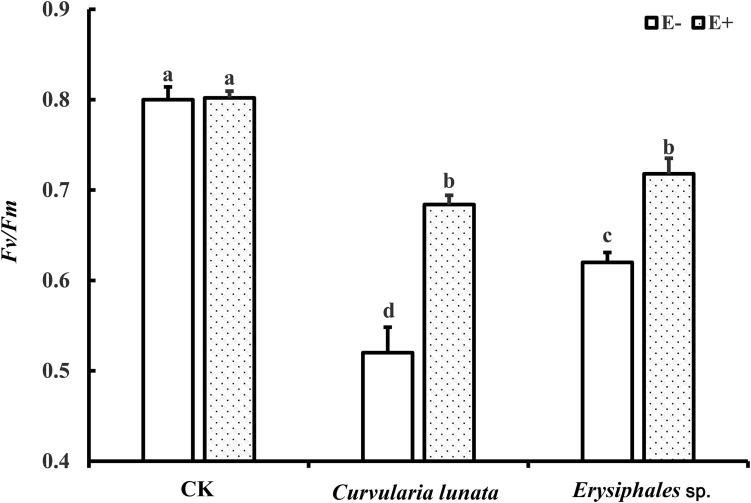
The *Fv/Fm* of E+ and E− *A. sibiricum* leaves infected by pathogens. Different lowercase letters indicate significant differences between treatments (*P* < 0.05). Bars represent mean values ± SE (*n* = 5).

The endophyte, pathogens, and their interaction all had significant effects on *Fv/Fm* (Table 1). In the control group without pathogens, the *Fv/Fm* of *A. sibiricum* leaves showed no significant difference between E+ and E− leaves (Figure 3). Pathogen inoculation significantly reduced the *Fv/Fm* of both E+ and E− leaves. For E− leaves, the adverse effect of *C. lunata* (reduced by 35% compared to that in CK) was significantly stronger than that of *Erysiphales* species (reduced by 23% compared to that in CK). The endophyte alleviated the decline in *Fv/Fm* in leaves infected by both pathogens. For E+ leaves, similar *Fv/Fm* values were observed in leaves infected by both pathogens, which suggested that the beneficial effect of endophyte infection was more significant when leaves were inoculated by *C. lunata* than when they were inoculated by *Erysiphales* species.

### Content of SA and JA

**FIGURE 4:**
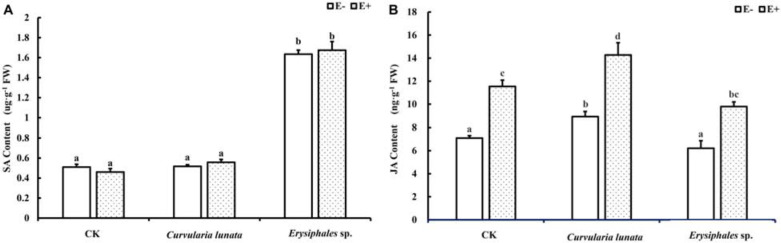
The salicylic acid **(A)** and jasmonic acid **(B)** content of E+ and E− *A. sibiricum* leaves infected by pathogens. Different lowercase letters indicate significant differences between treatments (*P* < 0.05). Bars represent mean values ± SE (*n* = 5).

Both endophytic fungi and pathogens significantly affected the content of JA in *A. sibiricum*, whereas the content of SA was only affected by pathogens (Table 1). There was no significant difference in the SA content between E+ and E− leaves in CK (Figure 4A), but the JA content in E+ was significantly higher than in E− (increased 63%) (Figure 4B). Compared with CK, inoculation by *C. lunata* did not affect the SA content in either E+ or E− leaves, but increased the JA content in both E+ and E− leaves, and the JA content in E+ was 60% higher than that in E−. When inoculated by *Erysiphales* species, the SA content was significantly increased in both E+ and E−, and there was no significant difference between E+ and E−. Inoculated by *Erysiphales* species did not affect the JA content in either E+ or E−, but the JA content in E+ was 58% higher than that in E−.

### Total Phenolic Compounds Content

**FIGURE 5:**
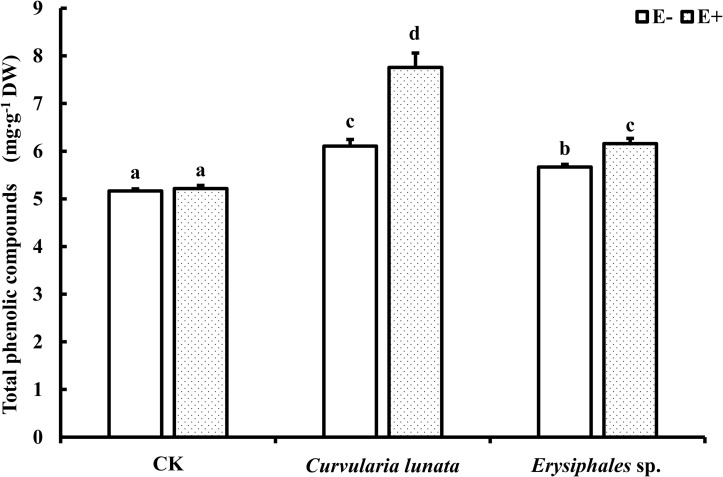
The total phenolic compounds content of E+ and E− *A. sibiricum* leaves infected by pathogens. Different lowercase letters indicate significant differences between treatments (*P* < 0.05). Bars represent mean values ± SE (*n* = 5).

The endophyte, pathogens, and their interaction significantly affected the content of total phenolics (Table 1). There was no significant difference in the total phenolic content between E+ and E− leaves in CK (Figure 5). Compared with CK, inoculation by the two pathogens caused a significant increase in the total phenolic content in leaves, and the total phenolic content of the leaves infected by *C. lunata* was higher than that of the leaves infected by *Erysiphales* species. The endophyte significantly increased the content of total phenolics in leaves infected by *C. lunata* and *Erysiphales* species by 27 and 8.7%, respectively.

## Discussion

Since Shimanuki and Sato (1983) first found that endophytic *Epichloë* could significantly reduce damage caused by *Blastocladia pringsheimii* in *Phleum pratense*, at least 15 grass–endophyte symbioses have been studied in the context of resistance to pathogens (Wiewióra et al., 2015; Xia et al., 2018). Most studies reported that *Epichloë* can improve the disease resistance of host, but several studies have found that endophytes have no effect on the disease resistance of the host or even have adverse effects (Welty et al., 1993; Wäli et al., 2006; Pańka et al., 2013a). Considering that pathogenic capacity differs among pathogen trophic types, necrotrophic pathogens are more destructive than biotrophic pathogens (Joanna et al., 2012; Kou and Naqvi, 2016). Therefore, is the effect of endophyte infection on host plant disease resistance related to the trophic types of pathogens? Previous research focused on pathogens of different trophic types by using different grass–*Epichloë* symbioses. For necrotrophic pathogenic fungi, the beneficial effects of endophyte infection have been reported in many grass species (Clarke et al., 2006; Ma et al., 2015; Wiewióra et al., 2015). For biotrophic pathogenic fungi, however, there are limited and varied results. Endophyte infection enhanced the resistance of *A. inebrians* to *Blumeria graminis* (Xia et al., 2015) and *Lolium multiflorum* to *Claviceps purpurea* (Perez et al., 2017), but had no effect on *F. pratensis* against *B. graminis* (Sabzalian et al., 2012).

The leaf spot diseases are widely distributed around northern China and can damage a variety of plants including grasses (Liu, 2011; Wei et al., 2011; Yue et al., 2019). In this study, we chose pathogens of different trophic types to inoculate the host in *A. sibiricum*–*Epichloë* symbiosis. The results showed that, compared to *Erysiphales* species, *C. lunata* caused a higher degree of damage and lower photochemical efficiency in E− leaves. The endophyte significantly alleviated the damage caused by these two pathogens to plant leaves. Similar *Fv/Fm* values and percentage of damaged leaf areas were observed between the two pathogen inoculation treatments in E+ leaves, indicating that the beneficial effects of the endophyte were greater against *C. lunata* than against *Erysiphales* species.

Salicylic acid is a plant signaling molecule that acts in response to biotrophic pathogens (White, 1979; Michael et al., 2018). In this study, we found that the content of SA in *A. sibiricum* leaves did not significantly change in response to inoculation by *C. lunata* but was significantly induced by *Erysiphales* species. The presence of endophytic fungi had no significant effect on the SA content in *A. sibiricum* leaves. In previous studies in our laboratory, Wang et al. (2016) also found that endophytes had no effect on the concentration of SA in *Leymus chinensis* infected by *C. lunata* or *Bipolaris sorokiniana*, indicating that the improvement of host disease resistance due to the endophyte was not regulated by SA.

Jasmonic acid is also an important signaling molecule in plant disease resistance responses. Many studies have shown that plants initiate JA−dependent responses upon exposure to necrotrophs (Mengiste, 2012; Qi et al., 2012; Pandey et al., 2016; Islam et al., 2018). In the present study, the content of JA in E+ and E− leaves was not affected by the biotrophic pathogen *Erysiphales* species but was increased by the necrotrophic pathogen *C. lunata*, which was consistent with the conclusions of previous research. In this study, we found that endophyte infection significantly increased the JA content in infected host grass leaves, regardless of which pathogen was inoculated, indicating that endophytic fungi may enhance the disease resistance of *A. sibiricum* hosts via the JA−mediated pathway.

Plants reportedly respond to inoculation of necrotrophic pathogens and biotrophic pathogens via the JA and SA pathways, respectively (Glazebrook, 2005; Vos et al., 2013), which was also demonstrated in this study. In this study, we further found that *Epichloë* enhanced the disease resistance of *A. sibiricum* host, probably via the JA−mediated pathway, not the SA pathway. Similar results have also been reported by Wang et al. (2016), who found endophyte infection enhanced the disease resistance of *L. chinensis* without affecting SA concentration of the host. Unfortunately, JA was not tested in that study. In contrast, Guo et al. (2019) found that endophyte infection improved the pathogen resistance of *L. perenne* but did not induce the increase of JA content. In addition, the effects of endophyte infection on SA/JA content have been reported by other studies, although their relationship with pathogen resistance was not considered. For example, *Epichloë occultans* significantly reduced SA concentrations but had no effect on the JA concentration in *L. multiflorum* (Bastías et al., 2018a, 2019). On the contrary, Ambrose et al. (2015) found that *Epichloë* endophyte in red fescue had a null or positive effect on SA concentration, depending on the plant tissue (leaf/sheath) and the endophyte. The diverse effects of endophyte infection on SA/JA concentration suggest that different pathogen resistance mechanisms might occur in different *Epichloë*–grass symbionts.

Phenolic compounds are important secondary metabolites in the plant disease resistance response (Cheynier et al., 2013). Plants can synthesize phenolic compounds such as tannins, coumaric acid, and ferulic acid to inhibit pathogenic activity (Blodgett et al., 2003; Yang and Gao, 2009; Bento et al., 2018) and produce lignin to resist pathogen invasion (Hématy et al., 2009). In the present study, the endophyte did not significantly affect the content of total phenolic compounds in the control but promoted the accumulation of total phenolic compounds in plant leaves regardless of which pathogen was inoculated. These results suggested that *Epichloë* could improve the disease resistance of *A. sibiricum* by promoting the synthesis of phenolic compounds. The enhancement of total phenolic compounds content of host grasses by endophyte infection to resist pathogens has also been reported in perennial ryegrass (Pańka et al., 2013a).

## Conclusion

Our study found that *A. sibiricum* initiated JA−related pathways to resist the necrotrophic pathogen *C. lunata*, while initiated SA−related pathways to resist the biotrophic pathogen *Erysiphales* species. *Epichloë* endophytes significantly alleviated the leaf damage caused by the two trophic types of pathogens, and the beneficial effects were more significant when hosts were exposed to *C. lunata* than when they were exposed to *Erysiphales* species. Endophytic fungi had no effect on the SA content but increased the content of JA and total phenolic compounds, which suggest that endophyte infection might probably enhanced the resistance of *A. sibiricum* to different trophic types of pathogens through similar pathways. It is worth noting that our results might have been different if different *Epichloë*–grass symbionts were studied, but the present study highlights that the interaction between the plant JA hormone and endophytes infection can affect the pathogen resistance of symbiotic plants.

## Data Availability Statement

The raw data supporting the conclusions of this article will be made available by the authors, without undue reservation, to any qualified researcher.

## Author Contributions

AR, XS, and YG designed the research. XS, MW, HL, TQ, JL and YS performed the experiments. XS and AR analyzed the data and wrote the manuscript. AR revised and polished the manuscript. All authors contributed to the article and approved the submitted version.

## Conflict of Interest

The authors declare that the research was conducted in the absence of any commercial or financial relationships that could be construed as a potential conflict of interest.
