# Corrigendum: Endophytic Fungi Activated Similar Defense Strategies of *Achnatherum sibiricum* Host to Different Trophic Types of Pathogens

**DOI:** 10.3389/fmicb.2020.611649

**Published:** 2021-02-05

**Authors:** Xinjian Shi, Tianzi Qin, Hui Liu, Man Wu, Juanjuan Li, Yansong Shi, Yubao Gao, Anzhi Ren

**Affiliations:** College of Life Sciences, Nankai University, Tianjin, China

**Keywords:** *Achnatherum sibiricum*, endophyte, jasmonic acid, pathogens, trophic type

In the original article, there was a mistake in Figure 4 as published. **The unit of JA content in** **Figure 4B** **should be “ng·g**^**−1**^**” instead of “ug·g**^**−1**^**”**. The corrected Figure 4 appears below.

**Figure 4:**
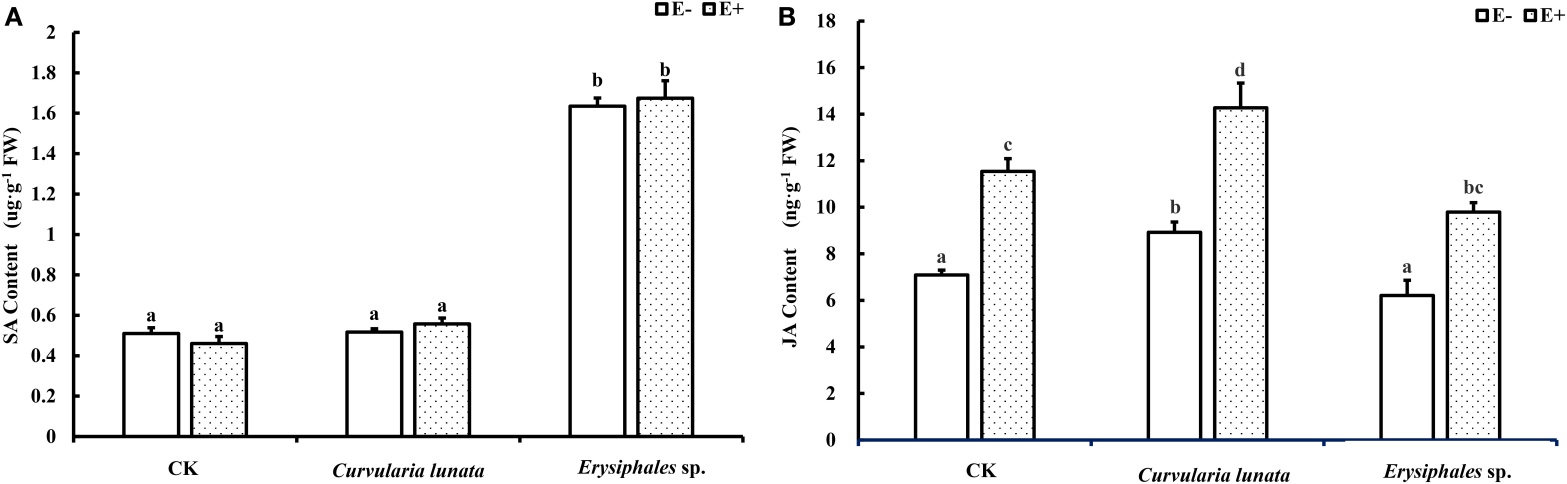
The salicylic acid **(A)** and jasmonic acid **(B)** content of E+ and E– *A. sibiricum* leaves infected by pathogens. Different lowercase letters indicate significant differences between treatments (*P* < 0.05). Bars represent mean values ± SE (*n* = 5).

The authors apologize for this error and state that this does not change the scientific conclusions of the article in any way. The original article has been updated.

